# Impact of Bone Cement Augmentation on the Fixation Strength of TFNA Blades and Screws

**DOI:** 10.3390/medicina57090899

**Published:** 2021-08-28

**Authors:** An Sermon, Ladina Hofmann-Fliri, Ivan Zderic, Yash Agarwal, Simon Scherrer, André Weber, Martin Altmann, Matthias Knobe, Markus Windolf, Boyko Gueorguiev

**Affiliations:** 1 Department of Traumatology, University Hospitals Gasthuisberg, 3000 Leuven, Belgium; an.sermon@uzleuven.be; 2 Department of Development and Regeneration, KU Leuven, 3000 Leuven, Belgium; 3 AO Research Institute Davos, 7270 Davos, Switzerland; ladina.hofmann@aofoundation.org (L.H.-F.); ivan.zderic@aofoundation.org (I.Z.); markus.windolf@aofoundation.org (M.W.); 4 SYNBONE SDN BHD, Kulai 81000, Malaysia; yash.agarwal@synbone.com; 5 DePuy Synthes Trauma, 4528 Zuchwil, Switzerland; sscherr1@its.jnj.com (S.S.); aweber9@its.jnj.com (A.W.); MALTMANN@ITS.JNJ.COM (M.A.); 6 Department of Trauma Surgery, Cantonal Hospital Lucerne, 6000 Lucerne, Switzerland; matthias.knobe@luks.ch

**Keywords:** hip fracture, osteoporosis, fracture fixation, augmentation, bone cement, polyurethane foam, bone surrogate

## Abstract

*Background and Objectives:* Hip fractures constitute the most debilitating complication of osteoporosis with steadily increasing incidences in the aging population. Their intramedullary nailing can be challenging because of poor anchorage in the osteoporotic femoral head. Cement augmentation of Proximal Femoral Nail Antirotation (PFNA) blades demonstrated promising results by enhancing cut-out resistance in proximal femoral fractures. The aim of this study was to assess the impact of augmentation on the fixation strength of TFN-ADVANCED^TM^ Proximal Femoral Nailing System (TFNA) blades and screws within the femoral head and compare its effect when they are implanted in centre or anteroposterior off-centre position. *Materials and Methods*: Eight groups were formed out of 96 polyurethane low-density foam specimens simulating isolated femoral heads with poor bone quality. The specimens in each group were implanted with either non-augmented or cement-augmented TFNA blades or screws in centre or anteroposterior off-centre positions, 7 mm anterior or posterior. Mechanical testing was performed under progressively increasing cyclic loading until failure, in setup simulating an unstable pertrochanteric fracture with a lack of posteromedial support and load sharing at the fracture gap. Varus-valgus and head rotation angles were monitored. A varus collapse of 5° or 10° head rotation was defined as a clinically relevant failure. *Results*: Failure load (N) for specimens with augmented TFNA head elements (screw/blade centre: 3799 ± 326/3228 ± 478; screw/blade off-centre: 2680 ± 182/2591 ± 244) was significantly higher compared with respective non-augmented specimens (screw/blade centre: 1593 ± 120/1489 ± 41; screw/blade off-centre: 515 ± 73/1018 ± 48), *p* < 0.001. For both non-augmented and augmented specimens failure load in the centre position was significantly higher compared with the respective off-centre positions, regardless of the head element type, *p* < 0.001. Augmented off-centre TFNA head elements had significantly higher failure load compared with non-augmented centrally placed implants, *p* < 0.001. *Conclusions:* Cement augmentation clearly enhances the fixation stability of TFNA blades and screws. Non-augmented blades outperformed screws in the anteroposterior off-centre position. Positioning of TFNA blades in the femoral head is more forgiving than TFNA screws in terms of failure load.

## 1. Introduction

The high morbidity and mortality rates of osteoporotic fracture patients are well-known. These fractures lead to an increasing percentage of hospital utilization for people over the age of 55 [1]. Due to the aging population, the socioeconomic impact of osteoporotic hip fractures becomes more relevant as more than 30% of the patients having sustained an osteoporotic hip fracture cannot return to their pre-fracture place of residence [2,3,4,5,6]. Despite the recent advances in implant design development, the reported rates of implant-related failures remain in the range of 2.0–16.5% [7,8]. Most of these failures are due to the underlying poor bone stock or cephalic implant malposition leading to cut-out and varus collapse [9]. These types of complications often require a reintervention and need to be avoided in an already vulnerable population of hip fracture patients. It is known that both fracture reduction and implant positioning play an important role in the prevention of complications [10]. The use of both tip-apex distance (TAD) and the more recently introduced calcar-related TAD demonstrate that eccentric (anterior, posterior or superior) placement of the head element (HE) might predict mechanical failures [11,12,13]. However, the clinical situation does not always allow for a perfect implant positioning and work on complications following hip fracture surgeries report that surgeons sometimes accept an eccentric HE placement [14].

On the other hand, there is an increasing interest in implant augmentation of the osteoporotic femoral head to enhance its cut-out resistance [15,16]. Previous biomechanical and clinical studies suggest that polymethylmethacrylate (PMMA)-based bone cements offer a significant improvement in the mechanical strength of the bone-implant interface, especially in presence of osteoporosis [17,18]. What remains unclear is whether one cephalic implant design is superior to another in the setting of cement augmentation and whether a situation with a non-ideally positioned implant can be salvaged by augmentation. 

Therefore, the aim of this study was to investigate systematically the biomechanical competence of two recently launched cephalic implants in an augmented and non-augmented state, inserted in either ideal (centre) or less ideal anteroposterior off-centre positions in simulated femoral heads with poor bone quality. It is hypothesized that the blade implants are biomechanically superior in all positions, and the addition of PMMA-based cement augmentation improves their construct stability in all tested conditions.

## 2. Materials and Methods

### 2.1. Specimens and Study Groups

A total of 96 surrogate specimens simulating human cancellous bone of femoral heads with poor bone quality, defined geometry (length 50 mm, width 38 mm, height 50 mm) and material properties (density 0.16 g/cm^3^ and elastic compression modulus 23 MPa) were manufactured of cellular polyurethane foam (#1522-10, 10pcf, Pacific Research Inc., Malmö, Sweden) as previously reported [19,20]. The specimens were assigned to eight study groups consisting of twelve specimens each (*n* = 12) and differing in blade or screw cephalic implantation, centre or anteroposterior off-centre implant positioning, and bone cement augmentation (yes, no) (Table 1).

### 2.2. Preparation

**Figure 1:**
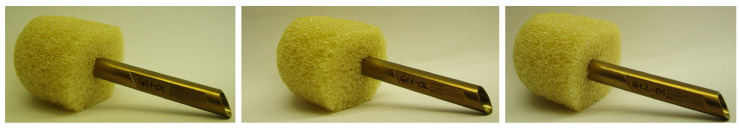
Photographs of surrogate specimens simulating human cancellous bone of right femoral heads implanted with TFN-ADVANCED^TM^ Proximal Femoral Nailing System (TFNA) head elements in central (**left**) and anteroposterior eccentric position with 7 mm anterior (**middle**) and 7 mm posterior (**right**) offset.

Each foam specimen was mounted in a custom-fabricated polymer shell (diameter of 56 mm) mimicking the femoral head cortex [19]. A perforated TFN-ADVANCED^TM^ Proximal Femoral Nailing System (TFNA, DePuy Synthes, Zuchwil, Switzerland) blade or screw HE of 100 mm length was implanted over a guidewire to a depth of 40 mm according to the manufacturer’s guidelines (Figure 1). 

During implantation, the anteroposterior eccentric implant position was defined by either 7 mm anterior or 7 mm posterior offset. Due to the specimen’s symmetry in the frontal plane, half of the specimens in each off-centre group were implanted with anterior or posterior offset. The resulting TAD in the centre and off-centre groups was 20 mm and 24 mm, respectively.

**Figure 2:**
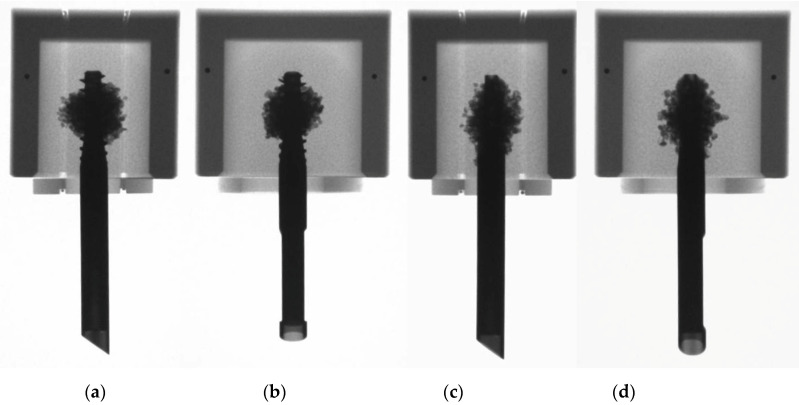
Radiographic images of cement-augmented specimens with centrally implanted TFN-ADVANCED^TM^ Proximal Femoral Nailing System (TFNA) screw/blade in anteroposterior (**a**,**c**) and superoinferior (**b**,**d**) views.

Implant augmentation was performed using a total of three ml PMMA-based bone cement (Traumacem V+, DePuy Synthes, Zuchwil, Switzerland) and following a standardized technique [19]. This cement amount corresponded well to the average injected volume in previous clinical work [21]. The distribution of the augmentation material was verified by means of radiographic images (Figure 2).

### 2.3. Mechanical Testing

**Figure 3:**
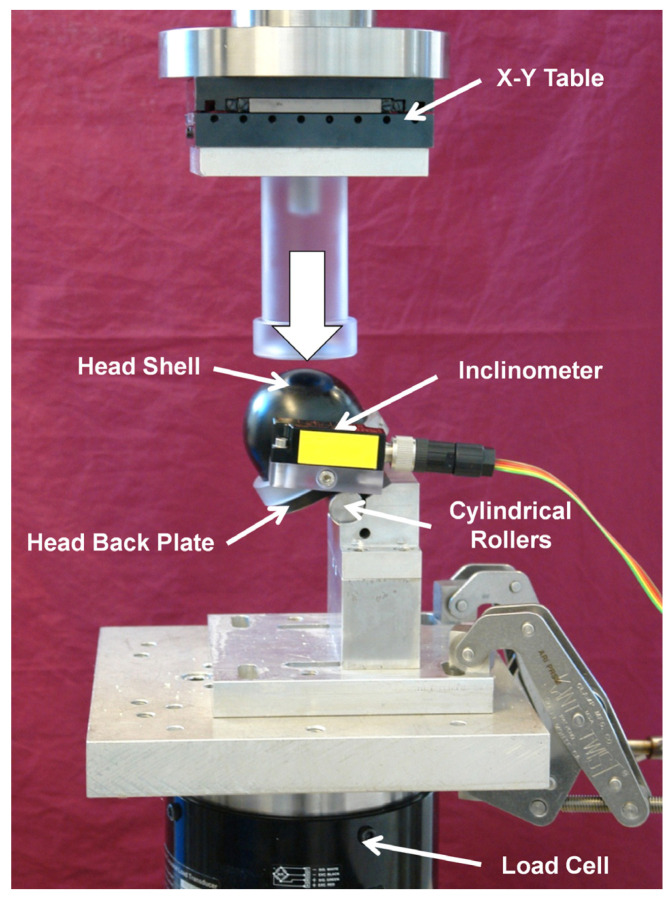
Setup with a specimen mounted for biomechanical testing. An unstable pertrochanteric fracture with lack of posteromedial support and load sharing at the fracture gap is simulated. Load transfer to the mimiced femoral head is performed with a physiological orientation. Each specimen is free to collapse in varus-valgus and rotation around the implant axes, both monitored by means of an inclinometer.

Mechanical testing was performed on a servo-hydraulic test system (Mini Bionix II 858, MTS Systems Corp., Eden Prairie, MN, USA) equipped with a 25 kN load cell. An adopted test setup from previous work was used to test each specimen by simulating an unstable pertrochanteric fracture with a lack of posteromedial support and load sharing at the fracture gap (Figure 3) [4,19,20]. 

Each specimen was mounted on a polycarbonate base plate resting on two cylindrical rollers allowing both varus collapse and rotation around the implant axis. The virtual intertrochanteric fracture line was located in a plane crossing the axis of the cylindric rollers. The HE was mounted to a base fixture, blocked in rotation and free to slide thus mimicking full implant dynamics, and inclined at 149° to the vertical axis, accounting for a situation of a femur with 130° femoral neck (caput-collum-diaphyseal) angle, oriented in 3° adduction and subjected to a load vector transferred to the femoral head in a physiological orientation of 16° to the vertical line [22,23]. 

Each specimen was loaded with a physiological orientation of the load vector via a spherical-shaped, greased shell, mounted on the machine crosshead actuator and simulating the acetabulum [20,24]. An inclinometer (Kübler Group GmbH, Villingen-Schwenningen, Germany) was attached to the specimen to monitor and record both varus-valgus and rotation around the implant axis angles throughout mechanical testing. 

All specimens underwent progressively increasing cyclic loading at 2 Hz. In order to simulate an alternating load during walking, an appropriate loading trajectory was derived from in vivo measurements in the human hip [22]. Starting at 400 N, the peak load of each cycle was monotonically increased by 0.1 N/cycle until failure of the bone-implant construct occurred. The valley load of the cycles was maintained at 100 N throughout the whole test. Testing was stopped when either 10 mm axial displacement of the machine actuator or 20° varus deformation occurred with respect to test initialization.

### 2.4. Data Acquisition and Evaluation

Machine data in terms of axial displacement (mm) and axial load (N), as well as inclinometer data in terms of varus-valgus and rotation around the implant axis (°) were simultaneously recorded at a rate of 128 Hz. Moreover, anteroposterior and superoinferior radiographs of each specimen were taken before and after testing to verify the adequate placement and augmentation of the HE, and monitor the failure mode, respectively. A varus collapse of 5° or a rotation around the implant axis of 10°, indicative for loosening of the implant, were defined as clinically relevant failure criterion, depending on whichever of these two events occurred first. The corresponding peak failure load was calculated for all specimens.

### 2.5. Statistical Analysis

Statistical analysis was performed using SPSS software package (IBM SPSS Statistics 27, SPSS Inc., Chicago, IL, USA). Normality of data distribution in each study group was screened and proved with the Shapiro–Wilk test. Univariate analysis of variance (ANOVA) with a Bonferroni post-hoc tests for multiple comparisons were performed to identify significant differences between the study groups. Level of significance was set to 0.05 for all statistical tests.

## 3. Results

**Figure 4:**
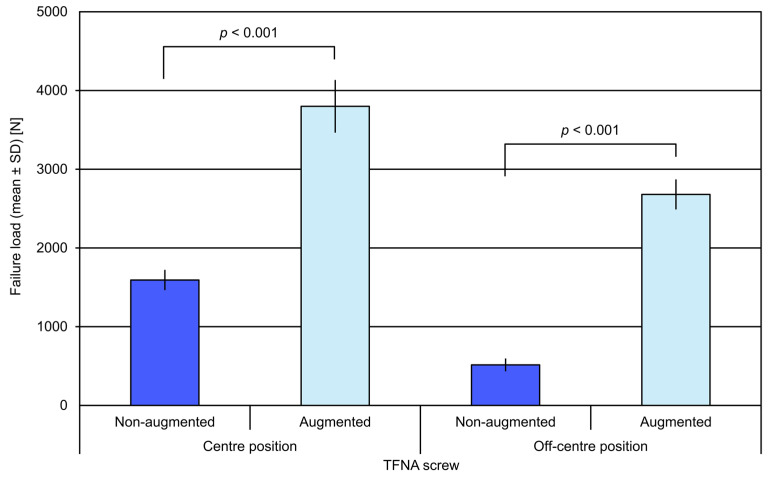
Failure load in the study groups with non-augmented and augmented TFN-ADVANCED^TM^ Proximal Femoral Nailing System (TFNA) screws, presented in terms of mean value and standard deviation (SD) together with *p*-values indicating selected significant differences.

The failure load for specimens with augmented TFNA screw head elements (screw centre: 3799 ± 326 N (mean ± standard deviation, SD); screw off-centre: 2680 ± 182 N) was significantly higher compared with the respective non-augmented specimens (screw centre: 1593 ± 120 N; screw off-centre: 515 ± 73 N), *p* < 0.001 (Figure 4).

**Figure 5:**
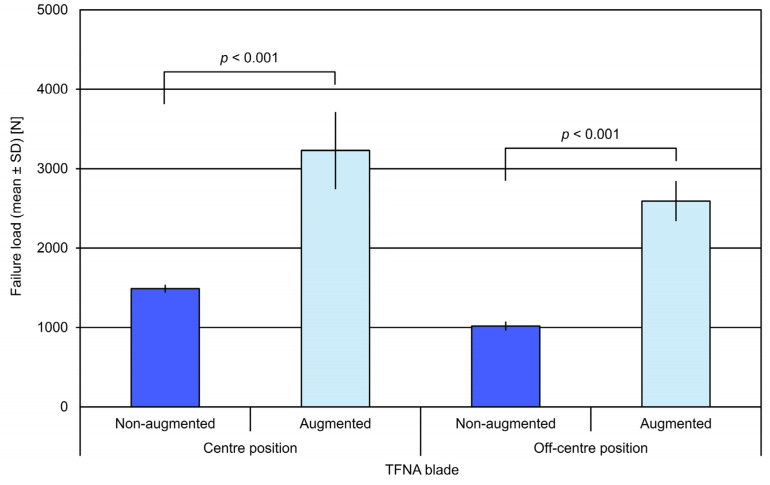
Failure load in the study groups with non-augmented and augmented TFN-ADVANCED^TM^ Proximal Femoral Nailing System (TFNA) blades, presented in terms of mean value and standard deviation (SD) together with *p*-values indicating selected significant differences.

Similarly, the failure load for specimens with augmented TFNA blade head elements (blade centre: 3228 ± 478 N; blade off-centre: 2591 ± 244 N) was significantly higher compared with the respective non-augmented specimens (blade centre: 1489 ± 41 N; blade off-centre: 1018 ± 48 N), *p* < 0.001 (Figure 5). 

**Figure 6:**
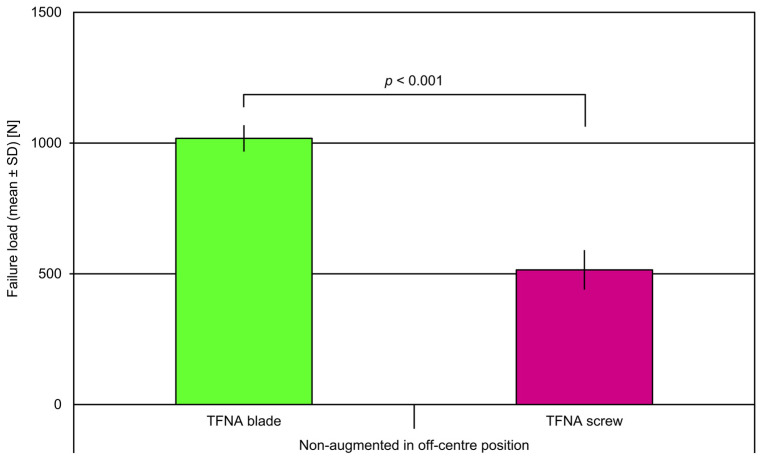
Failure load in the two groups with non-augmented TFN-ADVANCED^TM^ Proximal Femoral Nailing System (TFNA) blades and screws in off-centre position, presented in terms of mean value and standard deviation (SD) together with *p*-value indicating significant difference.

In both non-augmented and augmented specimens, the failure load in centre position was significantly higher compared with the respective off-centre position, regardless of the head element type, *p* < 0.001. Non-augmented TFNA blades in off-centre position revealed significantly higher failure load versus non-augmented screws in off-centre position, *p* < 0.001 (Figure 6). 

Augmented off-centre TFNA head elements had significantly higher failure load compared with non-augmented centrally placed implants, *p* < 0.001. No significant differences were found between anterior and posterior implant placement within any of the off-centre groups (*p* ≥ 0.327). No further significant differences were identified among the study groups.

In off-centre position, the TFNA screw demonstrated a 420% increase in failure load due to augmentation compared with a 155% increase for the TFNA blade. In centre position, the increase in failure load due to augmentation was 138% and 117% for the TFNA screw and blade, respectively (Table 2).

**Figure 7:**
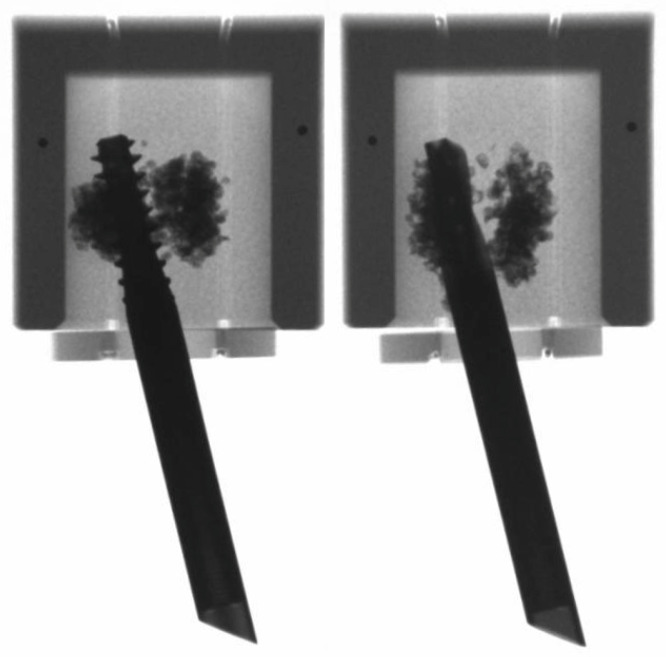
Mode of catastrophic failure for all augmented centrally implanted specimens featuring cement split into a cranial and a caudal segment.

All non-augmented and augmented centrally implanted specimens failed catastrophically by cut-out resulting in varus collapse. The cement was split into a cranial and a caudal segment for the augmented ones of them (Figure 7). In contrast, all non-augmented and augmented eccentrically implanted specimens failed catastrophically by rotation around the implant axis. No implant breakage was observed at any location of the HE in any of the tested specimens. No breakage at any location of the TFNA HEs was observed.

## 4. Discussion

This study supports the growing evidence that selected implant augmentation in the treatment of pertrochanteric fractures in poor bone quality adds a significant amount of stability to the bone-implant construct. The design of the perforated TFNA blade and screw allows an enhanced accurate injection of bone cement around the cephalic implant, effectively increasing the surface area of the bone-implant interface while minimizing the actual amount of cement required to achieve stability.

The findings of the current study are in agreement with previous clinical and biomechanical reports demonstrating the beneficial effect of cement augmentation on implant-to-bone anchorage by increased cut-out resistance [16,19,24,25,26,27]. In contrast, an investigation on the overall biomechanical performance of a screw-and-blade anchor implant system in a human cadaveric model with a simulated unstable femoral neck fracture concluded that cement augmentation results in no additional advantages [28]. Comparable biomechanical competence of the same screw-and-blade anchor implant system versus a helical blade and superiority versus a cephalic screw, all without cement augmentation, was reported in another study [29].

Improved cephalic implant design and meticulous application of the surgical technique resulted in safe and acceptable clinical outcomes [21]. Kammerlander et al. demonstrated in two prospective clinical trials that cement augmentation can safely be performed without evidence of joint extravasation, avascular necrosis of the femoral head, or radiographic chondrolysis at 15 months postoperatively [15].

One of the strengths of the current study is that it investigated systematically in well-defined laboratory conditions not only the two most commonly used HEs (blade and screw) but also explored the role of cement salvage in off-centre HE positions. The selected anteroposterior eccentric implant positioning, defined by either 7 mm anterior or 7 mm posterior offset, is in agreement with previous work on cephalic implant placement and seems to reflect well the reality in the surgical theatre [30]. The importance of minimizing the TAD of the sliding hip screw HE was well-established by the work of Baumgaertner and was extrapolated to several cephalic HEs in other studies; however, this ideal position is not always possible in complex clinical situations [11]. The blade HE was, in part, designed to increase the bone-implant surface interface and was shown more likely to avoid cut-out [31]. The current study supports the idea that the blade is more forgiving for malpositioning, especially in the non-augmented scenario. While there was no significant difference between the non-augmented blade and screw in the centre position, the non-augmented screw failed at significantly lower loads than the blade in the off-centre implantation. This suggests that in the setting of less-than-ideal implant positioning (revision surgery, patient anatomy, fracture reduction, etc.), a blade may be preferred over a screw.

The impact of cement augmentation on construct stability is the most interesting outcome of this study. The augmented groups, regardless of the implant position, demonstrated a homogeneous pattern of the cement distribution around the cephalic implants and significantly higher failure loads than their non-augmented counterparts. The dramatic improvement of 420% in the failure load of the off-centre TFNA screw by the addition of a small cement amount makes a strong argument to consider augmentation in the setting of a malpositioned screw that cannot be revised. However, it is very important to notice that the centre position of both cephalic implants in both augmented and non-augmented scenarios is the most stable. Therefore, this study fully supports the well-established mantra that proper implant positioning is of utmost importance.

The current study has several limitations, with the most notable related to the simulation of poor human cancellous bone quality by a foam material. Nevertheless, the foam material and model are well-established and have been used in multiple studies [19,20,32]. To avoid the limitations attributed to foam models, cadaveric bones can be used. Cadaveric bone offers a more life-like testing scenario, but studies have shown that testing results are also dependent of bone mineral density [25]. The foam model provides a more consistent testing situation. In addition, this study is limited by the incorporation of cephalic implants of only one manufacturing company. Several companies offer similar HE designs that might have different mechanical characteristics when tested. However, it is unlikely that these implants would differ enough to undermine the overwhelming outcome from this study that cement augmentation provides a significant benefit in femoral head specimens with poor bone quality.

## 5. Conclusions

Cement augmentation clearly enhances the fixation stability of TFNA blades and screws. Non-augmented blades outperformed screws in the anteroposterior off-centre position. Positioning of TFNA blades in the femoral head is more forgiving than TFNA screws in terms of failure load. The outcomes from this study should encourage orthopaedic trauma surgeons to consider the use of cement augmentation especially in patients with poor bone quality.

## Figures and Tables

**Table 1:** Groups in the current study, consisting of twelve specimens each and differing in cephalic implant type, implant position and cement augmentation.

Group Number	Implant Type	Position	Augmentation
1	Screw	Centre	No
2	Screw	Centre	Yes
3	Screw	Off-centre	No
4	Screw	Off-centre	Yes
5	Blade	Centre	No
6	Blade	Centre	Yes
7	Blade	Off-centre	No
8	Blade	Off-centre	Yes

**Table 2:** Increase in failure load for TFN-ADVANCED^TM^ Proximal Femoral Nailing System (TFNA) head elements (HE) due to cement augmentation as compared with the non-augmented specimen’s state.

TFNA HE	Failure Load Increase after Cement Augmentation [%]	
	Centre Position	Off-Centre Position
Blade	117	155
Screw	138	420

## Data Availability

Data availability upon request.

## References

[1] Singer A., Exuzides A., Spangler L., O’Malley C., Colby C., Johnston K., Agodoa I., Baker J., Kagan R. (2015). Burden of Illness for Osteoporotic Fractures Compared With Other Serious Diseases Among Postmenopausal Women in the United States. Mayo Clin. Proc..

[2] Friedman S.M., Mendelson D. (2014). Epidemiology of Fragility Fractures. Clin. Geriatr. Med..

[3] Papadimitriou N., Tsilidis K.K., Orfanos P., Benetou V., E Ntzani E., Soerjomataram I., Künn-Nelen A., Kymmer U.P., Eriksson S., Brenner H. (2017). Burden of hip fracture using disability-adjusted life-years: A pooled analysis of prospective cohorts in the CHANCES consortium. Lancet Public Health.

[4] Sermon A., Zderic I., Khatchadourian R., Scherrer S., Knobe M., Stoffel K., Gueorguiev B. (2021). Bone cement augmentation of femoral nail head elements increases their cut-out resistance in poor bone quality– A biomechanical study. J. Biomech..

[5] Svedbom A., Hernlund E., Ivergård M., Compston J., Cooper C., Stenmark J., McCloskey E.V., Jönsson B., Kanis J.A., EU Review Panel of IOF (2013). Osteoporosis in the European Union: A compendium of country-specific reports. Arch. Osteoporos..

[6] Vanhaecht K., Sermeus W., Peers J., Lodewijckx C., Deneckere S., Leigheb F., Boonen S., Sermon A., Boto P., Mendes R.V. (2012). The impact of care pathways for patients with proximal femur fracture: Rationale and design of a cluster-randomized controlled trial. BMC Health Serv. Res..

[7] Parker M.J., Handoll H. (2010). Gamma and other cephalocondylic intramedullary nails versus extramedullary implants for extracapsular hip fractures in adults. Cochrane Database Syst. Rev..

[8] Szita J., Cserháti P., Bosch U., Manninger J., Bodzay T., Fekete K. (2002). Intracapsular femoral neck fractures: The importance of early reduction and stable osteosynthesis. Injury.

[9] Bonnaire F., Zenker H., Lill C., Weber A.T., Linke B. (2004). Treatment strategies for proximal femur fractures in osteoporotic patients. Osteoporos. Int..

[10] Brunner A., Büttler M., Lehmann U., Curd Frei H., Kratter R., Di Lazzaro M., Scola A., Sermon A., Attal R. (2016). What is the optimal salvage procedure for cut-out after surgical fixation of trochanteric fractures with the PFNA or TFN?. A multicentre study. Injury.

[11] Baumgaertner M.R., Curtin S.L., Lindskog D.M., Keggi J.M. (1995). The value of the tip-apex distance in predicting failure of fixation of peritrochanteric fractures of the hip. J. Bone Joint. Surg. Am..

[12] Kuzyk P.R.T., Zdero R., Shah S., Olsen M., Waddell J.P., Schemitsch E.H. (2012). Femoral head lag screw position for cephalomedullary nails: A biomechanical analysis. J. Orthop. Trauma.

[13] Puthezhath K., Jayaprakash C. (2017). Is calcar referenced tip-apex distance a better predicting factor for cutting out in biaxial cephalomedullary nails than tip-apex distance?. J. Orthop. Surg..

[14] Tosounidis T.H., Castillo R., Kanakaris N.K., Giannoudis P.V. (2015). Common complications in hip fracture surgery: Tips/tricks and solutions to avoid them. Injury.

[15] Kammerlander C., Doshi H., Gebhard F., Scola A., Meier C., Linhart W., Garcia-Alonso M., Nistal J., Blauth M. (2013). Long-term results of the augmented PFNA: A prospective multicenter trial. Arch. Orthop. Trauma Surg..

[16] Kammerlander C., Hem E.S., Klopfer T., Gebhard F., Sermon A., Dietrich M., Bach O., Weil Y., Babst R., Blauth M. (2018). Cement augmentation of the Proximal Femoral Nail Antirotation (PFNA)—A multicentre randomized controlled trial. Injury.

[17] Röderer G., Scola A., Schmoelz W., Gebhard F., Windolf M., Hofmann-Fliri L. (2013). Biomechanical in vitro assessment of screw augmentation in locked plating of proximal humerus fractures. Injury.

[18] Wähnert D., Hofmann-Fliri L., Richards R.G., Gueorguiev B., Raschke M.J., Windolf M. (2014). Implant augmentation: Adding bone cement to improve the treatment of osteoporotic distal femur fractures: A biomechanical study using human cadaver bones. Medicine.

[19] Sermon A., Boner V., Schwieger K., Boger A., Boonen S., Broos P., Richards R., Windolf M. (2011). Biomechanical evaluation of bone-cement augmented Proximal Femoral Nail Antirotation blades in a polyurethane foam model with low density. Clin. Biomech..

[20] Sermon A., Hofmann-Fliri L., Richards R.G., Flamaing J., Windolf M. (2014). Cement augmentation of hip implants in osteoporotic bone: How much cement is needed and where should it go?. J. Orthop. Res..

[21] Kammerlander C., Gebhard F., Meier C., Lenich A., Linhart W., Clasbrummel B., Neubauer-Gartzke T., Garcia-Alonso M., Pavelka T., Blauth M. (2011). Standardised cement augmentation of the PFNA using a perforated blade: A new technique and preliminary clinical results. A prospective multicentre trial. Injury.

[22] Bergmann G., Deuretzbacher G., Heller M., Graichen F., Rohlmann A., Strauss J., Duda G. (2001). Hip contact forces and gait patterns from routine activities. J. Biomech..

[23] Sommers M.B., Roth C., Hall H., Kam B.C.C., Ehmke L.W., Krieg J.C., Madey S.M., Bottlang M. (2004). A Laboratory Model to Evaluate Cutout Resistance of Implants for Pertrochanteric Fracture Fixation. J. Orthop. Trauma.

[24] Sermon A., Boner V., Boger A., Schwieger K., Boonen S., Broos P.L., Richards R., Windolf M. (2012). Potential of polymethylmethacrylate cement-augmented helical proximal femoral nail antirotation blades to improve implant stability—A biomechanical investigation in human cadaveric femoral heads. J. Trauma Inj. Infect. Crit. Care.

[25] Erhart S., Schmoelz W., Blauth M., Lenich A. (2011). Biomechanical effect of bone cement augmentation on rotational stability and pull-out strength of the Proximal Femur Nail Antirotation™. Injury.

[26] Bartucci E.J., Gonzalez M.H., Cooperman D.R., I Freedberg H., Barmada R., Laros G.S. (1985). The effect of adjunctive methylmethacrylate on failures of fixation and function in patients with intertrochanteric fractures and osteoporosis. J. Bone Jt. Surg. Am..

[27] Von Der Linden P., Gisep A., Boner V., Windolf M., Appelt A., Suhm N. (2006). Biomechanical evaluation of a new augmentation method for enhanced screw fixation in osteoporotic proximal femoral fractures. J. Orthop. Res..

[28] Knobe M., Bettag S., Kammerlander C., Altgassen S., Maier K.-J., Nebelung S., Prescher A., Horst K., Pishnamaz M., Herren C. (2018). Is bone-cement augmentation of screw-anchor fixation systems superior in unstable femoral neck fractures? A biomechanical cadaveric study. Injury.

[29] Knobe M., Altgassen S., Maier K.-J., Gradl-Dietsch G., Kaczmarek C., Nebelung S., Klos K., Kim B.-S., Gueorguiev B., Horst K. (2017). Screw-blade fixation systems in Pauwels three femoral neck fractures: A biomechanical evaluation. Int. Orthop..

[30] Cleveland M., Bosworth D.M., Thompson F.R., Wilson H.J., Ishizuka T. (1959). A ten-year analysis of intertrochanteric fractures of the femur. J. Bone Jt. Surg. Am..

[31] Goffin J.M., Pankaj P., Simpson A.H.R.W., Seil R., Gerich T.G. (2013). Does bone compaction around the helical blade of a proximal femoral nail anti-rotation (PFNA) decrease the risk of cut-out?: A subject-specific computational study. Bone Jt. Res..

[32] Wähnert D., Lange J., Schulze M., Lenschow S., Stange R., Raschke M. (2013). The potential of implant augmentation in the treatment of osteoporotic distal femur fractures: A biomechanical study. Injury.

